# Global warming and mosquito-borne diseases in Africa: a narrative review

**DOI:** 10.11604/pamj.2023.44.70.37318

**Published:** 2023-02-06

**Authors:** Bachir Khezzani, Magdalena Baymakova, El Amine Khechekhouche, Ilia Tsachev

**Affiliations:** 1 Department of Biology, Faculty of Nature and Life Sciences, University of El Oued, PO Box 789 El-Oued 39000, Algeria,; 2 Laboratory of Biology, Environment and Health (LBEH), Faculty of Nature and Life Sciences, University of El Oued, PO Box 789 El Oued 39000, Algeria,; 3 Department of Infectious Diseases, Military Medical Academy, Sofia, Bulgaria,; 4 Department of Microbiology, Infectious and Parasitic Diseases, Faculty of Veterinary Medicine, Trakia University, Stara Zagora, Bulgaria

**Keywords:** Africa, global warming, mosquito-borne diseases, mosquitoes, public health

## Abstract

Human activity has a direct influence on the climate on our planet. In recent decades, the greater part of the scientific community has united around the concept of Global Warming (GW). This process highly impacts the geographical distribution of mosquitoes and Mosquito-Borne Diseases (MBD). The examined scientific publications show that Africa, especially sub-Saharan countries were and still hot spot of MBD globally. The economic, social, and environmental conditions prevailing in most African countries have effectively contributed to the spread of MBD. The current situation is very worrying, and it will get even more complicated as GW gets worse. In this regard, health systems in developing countries will have serious difficulties in health policies and public health activities to control the spread on MBD. Therefore, the governments of African countries should do more to combat MBD. However, a part of the responsibility lies with the international community, especially countries that contribute to GW. In conclusion, the analysis of the scientific literature showed that with increasing importance of GW leads to an increase in the prevalence of MBD.

## Introduction

Various human activities that have arisen and developed at an accelerating pace since the industrial revolution have imposed an important change on the atmosphere composition, both in quantity and quality [1,2]. Human activities have increased greenhouse gases concentration, such as nitrous oxide, chlorofluorocarbon (CFC), methane, and carbon dioxide in the Earth's atmosphere system [3,4]. These changes have led to an imbalance in the earth's heat budget. We are witnessing today a significant increase in the planet's temperature or which is called Global Warming (GW) [5]. El-Sayed and Kamel reported that GW is the greater challenge for mankind in the 21^st^ century because they threaten all aspects of our life [6]. The economic and social sectors, environment, and public health will be widely affected by GW [7].

The predictions show the mean global temperature will increase between 1.8°C (low scenario) and 4.0°C (high scenario) by the end of the 21^st^ century [8]. Africa is the continent most affected by the consequences of climate change despite producing only 2% to 3% of global greenhouse gas emissions [9]. In Africa, the temperature has increased by 0.7°C over the 20^th^ century. Also, it is predicted to increase further between 0.2°C and 0.5°C per decade [10].

All living things on this planet will be affected by climate change, and the change in their environment may lead to the extinction of many species [11]. However, the mosquito may benefit from GW [12]. The vital rates of these ectothermic vectors and pathogens are strongly affected by temperature (nonlinearly features), therefore, by GW [13]. A mosquito is a blood-sucking flying insect that serves as a vector, bringing dangerous pathogens and transferring them along to subsequent bite targets [14]. Excluding the areas permanently covered by ice, mosquitoes are found worldwide [15]. Mosquitoes are spread almost permanently in most tropical and subtropical regions and seasonally in other areas. Through their bite, mosquitos can transmit many dangerous diseases [14,16].

Mosquito-borne diseases (MBD) account for about 17.0% of the total burden of all infectious diseases [17], threatening over half of the world's population [18]. Every year, the World Health Organization (WHO) declares more than 300 million new incidence cases related to these diseases, of which more than 700,000 are lethal outcomes [19]. This situation poses a real threat to public health at the regional and global levels. The impacts of GW on MBD have caught a great deal of attention worldwide [20]. Furthermore, Ligsay *et al*. reported that the largest economic burden of disease in humans was borne by African and Asian countries [21].

## Methods

In writing this review, we used various inclusion and exclusion criteria. The inclusion criteria were as follows: (a) the analysis included original articles, short communications and case series; (b) studies were written in the English; (c) articles published between January 1950 and January 2022. The exclusion criteria were as follows: (a) the analysis excluded case reports, letters to the editor and surveys with missing data for the number of investigated individuals; (b) articles written in non-English (for example Arabic, Chinese, French, Russian or Spanish); (c) articles published before 1950. We searched the following databases for articles on the topic: PubMed, PubMed Central (PMC), Scopus, Web of Science and ScienceDirect. The last search date was September 12, 2022.

The databases search strategy included simple and combined keywords: “Africa”, “chikungunya fever”, “climate change”, “dengue fever”, “global warming”, “lymphatic filariasis”, “malaria”, “mosquito-borne diseases”, “Sub-Saharan Africa”, “vector-borne diseases”, “West Nile fever”, “yellow fever” and “Zika virus”. In the search process, filters were applied according to the accepted inclusion and exclusion criteria of this review. At data collection process and the analysis of the various elements of this data, the authors worked together by keeping in touch with each other through email, social media platforms (LinkedIn, Facebook, Twitter), and the applications for messages Telegram and Viber. In regard to study risk of bias assessment the researchers acted according to good practices of evidence-based medicine and their knowledge of infectious diseases. Of 205 examined peer-reviewed articles, 108 papers were excluded due to being irrelevant to the review topic. Finally, 97 peer-reviewed references were included for final review. The review of the scientific literature on the topic, we included in our review only articles that have a section.

**Ethical considerations:** all ethical rules and norms for working with humans and animals were observed during the conduct of the studies.

## Results

**Influence through vector and pathogens population:** mosquitoes are ectothermic organisms sensitive to external temperature variations that directly influence their body temperature [22]. However, Deichstetter report that the mosquito is the winner in the climate change challenge [11]. Several studies have addressed through laboratory experiments the potential effects of high temperature on the physiology, ecology and behaviours of both vectors and pathogens [23-25].

Alto and Juliano reported that high temperature (i.e. 30.0°C) was more favourable to adult development of *Aedes albopictus*, both in terms of time to adult emergence and the total number of individuals completing their development cycle [26]. Furthermore, Yang *et al*. have estimated through their mathematical model that *Ae. aegypti* mosquitoes need 29.2°C as the optimal temperature to produce the highest number of offspring and maintain a viable mosquito population [27]. The increased temperature allowed mosquitoes to breed faster through the increasing hatch, and development rates [23]. A study by Mourya *et al*. showed a significant increase in the susceptibility of *Ae. aegypti* to Chikungunya virus (CHIKV) with increasing temperature, which could be due to mosquitoes that overcame the higher temperature shock [28]. Singh and Purohit suggested that for every degree Celsius increase in global temperature, a latitudinal range of shift of about 200 km [4]. Also, An showed that an increase in temperature by 0.5°C led to increase of mosquito abundance by 30.0-100.0% [29].

According to the findings of Alto and Juliano, *Ae. albopictus* populations who live in areas with relatively high summer temperatures are expected to exhibit high population development rates, with adult populations peaking early in the season [23]. Increasing the temperature will accelerate the digestion process of the blood meal sucked by adult female mosquitoes, so the intensity of the suction will be higher. Also, it will accelerate the development process of the mosquito larvae into adults [30].

It was found that the optimal temperature interval for developing *Anopheles spp*. mosquitoes should be between 20.0°C and 30.0°C [31]. Therefore, in regions with less than optimal temperatures, mosquito survival will be increased if climate change raises temperatures to the optimum for the mosquito [32]. For example, *Ae. aegypti* has a thermal optimum of 29.0°C and can adapt well to the warmer microclimate, especially in urban environments. Furthermore, *Ae. albopictus* prefers a thermal optimum of 26.0°C and can be dormant in the temperate region during the cold winter; however, it loses its dormancy ability in warmer conditions [33]. Watts *et al*. reported that due to GW, the vectorial capacity increased by 27.6% for the malaria transmission in the highlands of Africa, 11.1% for *Ae. albopictus*, and 9.1% for *Ae. aegypti* compared to the 1950s baseline [34].

Many studies predict that GW will lead to sea-level rise [35]. A study by Ramasamy and Surendran carried out on tropical coasts has provided clues confirming that the breadth of brackish water bodies in coastal zones can increase the densities of salinity-tolerant mosquitoes such as *Culex sitiens* and *Anopheles sundaicus* [32]. On the other hand, it leads to an adaptation of freshwater mosquito vectors such as *Ae. aegypti, Ae. albopictus, An. culicifacies*, and *An. stephensi* to salinity.

Similar to the vector, GW can affect pathogens. Higher temperatures help *Plasmodium spp*. to multiply quickly inside the vector; also, it decreases the Dengue virus (DENV) incubation time and increases the transmission rate [8,36]. A research by Lardeux and Cheffort in French Polynesia found that high temperature led to a significant decrease in the extrinsic incubation periods of *Wuchereria bancrofti* and an increase in the number of infective bites in *Ae. polynesiensis* [37]. Also, an reported that higher temperatures lead to shortens the extrinsic incubation period to less than 10 days for *P. falciparum* and *P. vivax*, which leads to more malaria transmission [29]. Reinhold *et al*. showed that high temperature decreased the extrinsic incubation period in *Ae. aegypti*; which could not transmit the Yellow fever (YF) virus at 18.0°C, while it was able to transmit it only four days after infection at 37.0°C [38]. Liu *et al*. reported that at 18.0°C, DENV-2 was confined to the midguts of *Ae. albopictus*, but between 23.0°C and 32.0°C, DENV-2 was able to invade the salivary glands [39]. Also, the survey found higher transmission rates and shorter extrinsic incubation periods with higher temperatures. In general, World Health Organization (WHO) reports estimated that about twenty neglected tropical diseases, especially Dengue fever (DENF), can re-emerge in developed countries due to GW [6].

Although most studies indicate that GW will be a cause of an increase in MBD, other surveys suggest the exact opposite [40]. A research by Paaijmans *et al*. on rodent malaria reported that vector competence tails off at higher temperatures, even though the parasite development rate increases [24]. Furthermore, in some regions where MBD risk is high, recent projections based on a monomodal relationship with temperature predict that future warming will reduce transmission risks, where temperatures will exceed optimal and marginal temperature limits for mosquitoes [41,42]. A predictive model by Mordecai *et al*. has shown that optimal malaria transmission occurs at 25.0°C; however, it decreases at a temperature above 28.0°C [43]. Reiter referred that the histories of YF, DENF and malaria show that climate has rarely been the principal factor determining their distribution; in contrast, human activities’ impact on local ecology has generally been much more significant [44]. Also, the same reference reported that the use of climate-based models to predict future prevalence considering inappropriate. According to several studies, the effects of temperature on MBD cannot simply be projected based on its known effects on vector and parasite parameters in the laboratory [13,25].

**Influence through human activities and behaviours:** human behaviours influenced by temperature can increase MBD outbreak [29]. However, humans bear a large part of the responsibility for the incidence of these diseases by ignoring the necessary precautions. In addition, an increase in temperature at the daily, monthly, or annual level will inevitably change humans´ behaviours. The locals go to ponds and swamps of stagnant water, whether for water supply or cooling. Therefore, it is inevitable that the infection will increase because these areas are considered natural habitats for mosquitoes. In addition, there will be an escape from stifling urban and semi-urban areas to jungles and forests, which will enhance the contact between vectors and humans. In rural areas, the villagers move to the highlands, the preferred habitat for some types of mosquitoes that do not exist in urban areas, which enhances and complex the infection cycle. Because of the heat, the population is forced to sleep outside the protected rooms and minimize wearing clothes, which increases the body area exposed to the bite, especially in the male gender. So, the probability of being bitten by mosquitoes is high [45]. In the heat season, some rural populations favourite to sleep outside or in woody houses because they are cooler than houses built with metals or cement, where they will be more likely to be bitten by infected mosquitoes [29,46].

Furthermore, human communities within the current geographic range of the mosquito-spread have some experience in the control and prevention of MBD. In contrast, other communities that fall under the threat of a new warming scenario will not be able to contain the situation in a short period because the disease endemicity is insufficient to provide a good protective immunity [32]; also, health systems are not adequately ready. Therefore, the consequences of mosquitoes spreading in new areas will be disastrous for public health. Patients, especially in rural areas, often opt for treatment from traditional healers instead of travelling long distances for medical care [47]; however, poverty can explain this behaviour. In Henan province, China the behaviour of the local population was a cause of a malaria outbreak [48]. Although the availability of bed nets, they did not offer complete protection because outdoor sleeping and staying up late were widespread behaviour during the hot summer [48].

**Influence through economic, social and environmental factors:** it is known that GW will lead to frequent and widespread processes such as drought, desertification, deforestation, floods, loss of agricultural productivity, and lands degradation [15]. In a continent such as Africa, GW will be disastrous to natural resources and worn health systems in origin.

**Economic and social factors:** in addition to GW, the (re)-emergence of MBD is attributed to a group of other combined factors [18]. However, economic and social factors are widely recognised as significant drivers of MBD risk [16]. Economic and social conditions may limit the capacity of public health systems to respond to changes that can occur because of GW on the level of MBD [49]. The economic situation of many African countries reflects on weak budgets allocated to the health sector [50]. Therefore, a wide category of sub-Saharan African residents does not have access to medical care because of poverty [51]. According to Watts *et al*. Africa showed a significant decrease in indexes related to health and climate change, where the decline was 8.4% for the surveillance capacity score and 6.8% for the response capacity score [34]. So, the lack of accessible diagnostic reagents with suitable prices in Africa is an obstruction to systematic surveillance, which leads to underestimation and underreporting of MBD [47].

Global warming is likely to affect public health through food insecurity and malnutrition outbreak, which leads to increasing in risk areas for MBD [52]. Poverty is a key social determinant in the MBD outbreak, especially, through its social expression as bad environmental and housing conditions [53]. A logistic regression model using Gross Domestic Product per capita (GDPpc), precipitation, and temperature found that about 5.2 bln. population will be at malaria risk by 2050 when considering climatic effects only. However, this number is decreasing to 1.95 bln. when considering the interaction between effects of climate and GDP and to 1.74 bln. when considering GDP effects only [54]. So, in light of these expectations, it can be said that the increase in GDPpc can reduce the negative effects of GW on the malaria burden. In poverty communities, most houses are made of thatch and wood, which don't provide their occupants full protection against mosquitoes´ bitts [29,48]. A research in Burkina Faso reported that children who lived in iron-sheet roofed houses had a much lower risk of getting *P. falciparum* infection than those who lived in traditional homes with mud roofs [46]. So, economic and social development and improvements to housing quality are also predicted to will promoted reductions in MBD transmission [46]. For example, a recent survey in Gambia showed that raising houses by 3 m above ground level reduces *An. gambiae* entry into the residence by 84.0% compared with huts on the ground [55].

**Precipitation:** the change in precipitation and humidity are climatic factors that can accompany the increasing temperature, and also plays important roles in mosquito population dynamics because warmer air holds more moisture and encourages mosquito survival [29,32]. The records of precipitation from 1951 to 2010 showed that there had been a significant increase in Central, Eastern, and Southern Africa [56]. By the end 21^st^ century, and under the high greenhouse gas emission scenario, yearly precipitation is predicted to increase by 5.0% to 75.0% in Eastern Africa. In contrast, it is expected to decrease by 15.0% across Western Africa and between 15.0% and 45.0% in Southern Africa [8,57]. For example, precipitation has declined by 50.0% in Niger and Senegal [58].

It is evident that precipitation patterns will affect MBD in both cases, drought or inundation. Precipitation contributes to the creation and continuation of new mosquito breeding habitats, thus increasing the vector population, especially in immature stages, while the rise in humidity is more suitable for adult mosquitoes' survival [59,60]. However, excessive rainfall can wash larvae and eggs away and reduce the number of small puddles, temporarily lowering mosquito populations [32]. Because the quality of the breeding water is an important factor in the survival of mosquito eggs, high precipitation contributes to improving the quality of water in valleys and swamps and leads to more speared of mosquitos [61]. This viewpoint is consistent with what Alto and Juliano found in their experiment that simulated the interaction between the increase in temperature and precipitation values that have led to increased production of mosquito adults [26]. In African coastal zones, higher rainfall can extend the sites of fresh water vectors such *An. gambiae* [62]. A study in Zambia reported that for an increase by 1mm in rainfall and 10.0% in humidity, there is an increase in malaria infection of about 0.2% and 2.0%, respectively [63].

In case of drought, increased stress may encourage water storage practices that increase proximity to mosquito habitat, especially *Ae. albopictus* and *Ae. aegypti* live closely associated with human populations and prefer to breed in domestic water containers [64,65]. During the DENF outbreaks in Kenya in 2014 and Sudan in 2010, the investigations found different categories of open water storage tanks that provide favorable breeding sites for mosquitos in all affected households [47,66].

The decrease in precipitation leads to the drying up of rivers and the formation of pools in river beds, which in turn helps create new suitable sites for mosquitos, and makes the larval populations prove disadvantageous to predators [4,29]. In addition, the rural communities are forced to move to search for water, which leads to more contact between the host and the mosquito in its environment. According to Khasnis and Nettleman, disease transmission may be enhanced through the scarcity and contamination of potable water sources; furthermore, crops fiasco and famine may diminish host resistance to infections [67]. On the other hand, in African coastal areas is that a drier climate can favor salinity-tolerant vectors, like *An. merus* and *An. melas* [32]. On the other hand, the change in precipitation will contribute to the spread of waterborne diseases, which, in turn, complicates the health status of the affected areas and decreases the immunity of the population [58].

**Land-use change:** land-use change is the process through which human activities alter the natural environment. It refers to how the land has been used, with an emphasis on its functional role in economic activities [68]. Land-use change occurs in response to rapid population growth, intensification of agricultural activities, expansion of cities and the realisation of grand infrastructure projects. It can also occur due to climate change [69,70]. Land-use change is likely to increase due to GW, and the latter is an important cause for the occurrence of the first [70]. The distribution of mosquito vectors and pathogens can be affected by changes made by humans in natural environments, such as irrigation projects and intensive agricultural activities; through increasing the environmental suitability for the vectors development and the susceptible populations density [49].

A research on *An. gambiae* and *P. falciparum* realised by Lindblade *et al*. in a highland area of Uganda showed that land-use change by replacing natural swamp vegetation with agricultural crops has led to increase both maximum and minimum temperatures by approximately 0.9°C; which in turn led to a rising incidence rate of malaria [71]. Furthermore, in Kenya Afrane *et al*. reported that the gonotrophic cycle of female *An. gambiae* was shortened by 21.0% (2.9 days) and 52.0% (2.6 days) during the rainy and dry seasons, respectively, in open deforested areas compared with forested sites [72].

A survey by Ghebreyesus *et al*. has shown micro dams established in Northern Ethiopia led to an increase in malaria incidence sevenfold in villages within 3km compared with control villages 8-10 km distant [73]. In 1990s, the irrigation projects improved breeding conditions for *An. culicifacies* in India. As a result, malaria became endemic and widespread in roughly 200 mln. People [74]. In Paraguay, Wayant *et al*. found that incidence of malaria was associated with deforestation process [75]. After the Aswan High Dam construction (Aswan city, Egypt), the prevalence of lymphatic filariasis in Egypt increased from 1.0% in 1965 to 20.0%. The new conditions created and improved breeding sites for *Cx. pipiens* [76]. In the same context, a study in Ghana found that annual bites per person, annual transmission rates and infection rates related to *An. gambiae* were higher in irrigated regions than without irrigation regions [77]. In short, analysis of land-use change due to GW and its effects on habitat and biodiversity is an important process for MBD risk assessments.

**Urbanisation:** over the past three decades, most urbanisation has occurred in developing countries. The global population residing in urban areas increased from 34.0% in 1960 to reach 54.0% in 2016; moreover, it is predicted to increase to 2.5 bln. by 2050, predominantly in Asia and Africa [16]. The urban population in sub-Saharan Africa has increased from 10.0% in 1950 to 35.0% in 2013, with more than 50.0% anticipated by 2030 [78]. African countries suffer from severe infrastructure deficits compared of the population growth [79]. Due to economic and social factors, urbanization in most African countries is unplanned, furthermore GW promotes unplanned urbanisation [80]. This type of urbanization is a major factor in the increasing mosquito populations. Accumulation of nonbiodegradable containers in and around living areas has provided a micro aquatic environment suitable for mosquito proliferation [81]. Slum populations face a high spread of MBD. Indeed, poor sanitation infrastructure and stagnant water can help mosquitoes breed, while limited protection and high population density promote human transmission [79].

In many parts of the world, the urbanisation was the major factor for DENF transmission through the unique opportunities offered to the mosquitoes by urban environments [82]. *Ae. albopictus* prefers to develop its larvae in water collections outdoors; therefore, more dependent on rain-fed habitats such as water collections in discarded containers, tree holes, and leaf axils. Furthermore, it considers the alternative DENF vector, especially in peri-urban and rural areas [32]. Also, about 79.0% of mosquitoes bites occur indoors at night [55]. Franklinos *et al*. report that unplanned urbanization was a major cause for the failure of many public health programs [16]. For example, the poor sanitation in Mandera City (Kenya) was the principal cause of the DENF outbreak spread in 2011, which led to 5,000 incidence DENF cases [47]. An experiment in Western Kenya highlands shows that the survivorship of *An. gambiae* in sunlit artificial pools (open habitat) was high at about 50.0% compared to the forested areas [83]. In epidemic areas, improving housing conditions is an urgent necessity to combat insect-borne diseases [84]. A research by Tusting *et al*. found that housing is an important risk factor for malaria; further, residents live in modern homes had 47.0% lower odds of malaria infection compared to those live in traditional homes [85].

## Discussion

**What strategy does Africa need in combating mosquito-borne diseases and global warming impacts?** Africa is highly vulnerable to GW; however, there is a consensus that most African countries are still groaning under the burden of MBD. This point of view has been confirmed by most academic studies and global health reports [19,86]. It is clear that the preparations for the predicted effects of GW are woefully inadequate, which will lead to worsening the damage. The ability of humanity to react or adapt depends on the magnitude and speed of the change. The outcome will also depend on our ability to recognize epidemics early, contain them effectively, and commit appropriate resources to treatment, prevention, and research [67]. Time is an important factor in reducing the imminent dangers of GW on MBD dynamics. In this regard, it would be beneficial to take a decisive step by preparing human communities for the upcoming climate scenario by all possible means and ways. According to Deichstetter personal protection and vector control are the principal two ways to reduce MBD transmission [11]. The following recommendations can help in achieving this purpose:

First, recent research by Saleh *et al*. in Zanzibar (Tanzania) showed that the preparedness of the healthcare system for timely detection, management, and control of various MBD outbreaks was critically low [87]. Furthermore, the same applies to most African countries. It is certain that GW will complicate the situation and increase pressure on health centers. Therefore, it is necessary to expedite the strengthening and rehabilitation of the health systems of African countries, which should be more resilient to succeed in managing health risks. Second, in Africa, most training workshops for health workers are in outbreak periods and for a short time with little or no impact [47]. Therefore, it is necessary to organize training workshops periodically to update knowledge and skills on appropriate diagnostic testing and disease reporting mechanisms; with the participation of experts from various fields such as databases management, entomology, diagnosis, and geospatial analysis. Third, several studies have proven the effectiveness of bed nets, especially insecticide-impregnated bed nets, in combating mosquitoes; however, governments and health authorities must provide them permanently, free of charge, or at nominal prices, particularly during mosquito peak spread [88,89].

Fourth, educational and awareness programs addressed to local communities are important tools in achieving the goals set for any policy. According to a survey conducted in Pakistan, community inaction or indifference to government programs aimed to eliminate mosquito nests increased of epidemic conditions [90]. Such behavior is likely to be common in African communities. Therefore, intensifying awareness campaigns on the prevention of MBD should be quickly activated by all possible means. It is certain that widely publicized awareness programs will lead to behavioral change and promote personal and community protection against MBD. Fifth, humans bear a large part of the responsibility in protecting their selves by wearing protective clothing, covering water tanks, cleaning the living environment, not sleeping outdoors, and reporting suspected cases. Sixth, although mechanisms related to GW that will influence MBD patterns are understood, future changes are difficult to predict, as is the case in most studies, because of the complexity of host, pathogen, vector, and environment relationships. Therefore, it is useful that the adoption of predictive models is not limited only to climatic factors, but special attention should be given to the rest of the factors contributing to the accuracy of the results obtained, especially if it comes to Africa. In this context, Beck-Johnson *et al*. confirm the importance of including mosquito biology in predictive models of MBD [91]; while Tozan *et al*. focus on the inclusion of social and economic development trajectories that permit for a more realistic assessment of the MBD risks in GW future scenarios [49].

Seventh, managing and eliminating preimaginal development sites through insecticide applications are important components of MBD control programs. However, because of the restrictions on traditional insecticide-based strategies, particularly the development of insecticide resistance, the adoption of biological control must be considered. In this context, many studies, such as Elias *et al*. and Benelli *et al*., have suggested biological alternatives to reduce the risks of using chemical pesticides [92,93]. Eight, individual or local efforts to combat MBD often fail, so it is necessary to coordinate efforts and develop unified vector-control strategies in African countries [94]. Furthermore, there must be harmony between strategies implemented by the governments and strategies developed by academics to achieve the highest effectiveness in combating MBD. In addition, eliminating MBD may be the long-term goal of control programs, while it must remain the priority in the short and medium-term. However, the intervention policies should be suitable for the local environment because the epidemiology, pathogens transmission intensity, and vector ecology are highly geographically variable.

Ninth, researchers, and scientists should share their experiences and provide policymakers with relevant information to develop suitable plans to build resilience to main MBD in a warmer world. Tenth, currently it is difficult to eradicate MBD in Africa, but it is not impossible. For example, Badmos *et al*. point out the possibility of benefiting from the Chinese experience, which was able to eradicate malaria completely [95]. Also, Europe has officially eliminated malaria since 1975 via the chemical treatment of patients, the drainage of wetland and stagnant water, improvement of the water system and sewage infrastructure, and biological control of the mosquito larvae using the fish Gambusia holbrooki [96]. Eleventh, some studies indicate that there is a mismanagement of international aid directed to Africa in the context of MBD control. Therefore, the optimal use of donations and limited resources (especially in low-income countries) is a big challenge to delivering the highest public health impact.

The twelfth, problem that restrains the eradication of MBD efforts in African countries, is the difficulty in tracking and notification. Recent research in Indonesia by Ramadona *et al*. demonstrated the role of social media data in tracking DENF spread in the intra-urban by weighting local incidence data with geotagged “Twitter” data as a proxy for mobility patterns across 45 neighborhoods in Yogyakarta city [97]. This novel approach is a low-cost source and can be used in African countries on a large scale to compensate for the shortfall in MBD surveillance and reporting in real time. Thirteenth, perhaps there is not much that African countries can do to mitigate the effects of GW, as a large part of the responsibility lies with the major industrialized countries. For example, a recent study reported that reducing GW to 1.5°C might avoid about 3.3 mln. Dengue fever cases per year in Latin America compared to no intervention (+3.7°C) [20]. The experience of the spread of SARS-CoV-2 infection has taught us that no community can, whatever they do, live in isolation. So, by applying the principle “you help others protect themselves = you protect yourself”, developed countries with a high level of scientific research and high biotechnology can help African countries.

## Conclusion

Global warming has earnest implications for all aspects of human life, including vector-borne diseases; however, mosquitos are a winner in the GW challenge. This literature review indicated that Africa is highly vulnerable to GW; however, the preparations for the predicted effects of GW are woefully inadequate, worsening the damage. Mosquito-borne diseases were and still are the major threat to public health, especially in sub-Saharan Africa. The situation of MBD and the factors that control them is very complex in African countries. In addition to the changing climatic factors, economic, social, and political conditions play an important role in determining the spread of these diseases, which their contribution in many cases exceeds the contribution of climatic factors. The governments of African countries should exert more effort to combat MBD by rehabilitating health systems and improving the economic, social, and environmental conditions of their populations. However, more research is needed to deepen our understanding of MBD dynamics and provide policymakers with relevant data to prepare appropriate strategies to increase flexibility to combat these diseases in a warmer world.

### What is known about this topic

- *Africa is a hotspot of mosquito-borne diseases outbreak;*
- *Infection risk with mosquito-borne diseases is controlled by a complex set of demographic, social, and environmental factors;*
- *Mosquito-borne diseases prevent development efforts in many African countries*.

### What this study adds

- *Global warming will exacerbate the situation of mosquito-borne diseases in Africa;*
- *African governments should benefit from the experiences of countries that have eradicated mosquito-borne diseases;*
- *Part of the responsibility for the mosquito-borne diseases outbreak lies with the international community, especially countries that contribute to global warming*.
